# Prevalence and Associated Factors of Smoking Pattern Among Adult Women and Men in Nepal: Evidence From Demographic and Health Survey 2022

**DOI:** 10.1002/puh2.70321

**Published:** 2026-07-30

**Authors:** Om Chandra Thasineku, Yogendra Bahadur Gurung, Sudesh Pandit

**Affiliations:** ^1^ Research Centre for Educational Innovation and Development Tribhuvan University Kathmandu Nepal; ^2^ Tribhuvan University Kathmandu Nepal; ^3^ Prithvi Narayan Campus Tribhuvan University Pokhara Nepal

**Keywords:** adults, men, public health, smoking pattern, women

## Abstract

**Introduction:**

Smoking in Nepal poses significant public health challenges linked to chronic cardiovascular and respiratory disease and socioeconomic consequences. This study aims to examine the prevalence and associated factors of smoking patterns among Nepali adults using national representative data.

**Methods:**

The secondary data from the Nepal Demographic and Health Survey 2022 were the source of data for analysis. A complex sample analysis was conducted to accommodate the stratified and cluster design used in the survey with sampling weights. Multivariable binary logistic regression analysis was carried out to identify significant predictors.

**Results:**

The prevalence of smoking patterns was 3.8% among 14,845 women and 28.0% among 4,913 men. Among women from Karnali province (adjusted odds ratio [AOR] = 3.144; *p* = 0.007), Janajati (AOR = 4.080; *p* = 0.003), women with no education (AOR = 3.705; *p* = 0.000), the 35–49 age group (AOR = 18.258; *p* = 0.000), and alcohol users (AOR = 2.808; *p* = 0.000), similarly, among men from Koshi (AOR = 1.881; *p* = 0.000), Dalit (AOR = 1.402; *p* = 0.030), men with basic education (AOR = 1.704; *p* = 0.001), the 20–34 age group (AOR = 1.597; *p* = 0.002), those engaged in nonagriculture (AOR = 2.430; *p* = 0.000), and alcohol users (AOR = 3.236; *p* = 0.000) were found more likely to smoke compared to their corresponding reference categories.

**Conclusions:**

Smoking among adults in Nepal is shaped by structural and sociocultural factors, such as province, caste/ethnic groups, educational attainment, occupational status, age and alcohol consumption. The difference between women and men reveals the necessity of equity‐oriented smoking control program and strategies that address the structural and sociocultural factors of smoking on the higher level and promote smoking cessation through combination with public health and social development programs.

## Graphical abstract

The prevalence of smoking in Nepal remains substantially higher among men than women and is strongly associated with province, caste/ethnicity, education, occupation, age, and alcohol consumption. These findings highlight the need for equity‐oriented and socioculturally responsive Tabacco control strategies in Nepal.

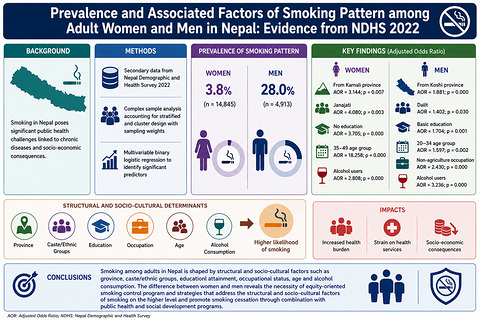

## 1 Introduction

Around the world, smoking continues to be a significant issue for public health and remains a major contributor to many preventable causes of morbidity and premature mortality [1]. Smoking is one of the leading avoidable risk factors for a wide range of cardiovascular diseases, chronic respiratory diseases, and many forms of cancer [2], accounting for avoidable social and economic burdens for households and healthcare systems [3]. The recent Nepal Demographic and Health Survey (NDHS) report 2022 indicates that 28.0% of men and about 3.8% of women aged 15–49 years in Nepal currently smoke, indicating an ongoing public health issue and continuing inequity in terms of tobacco consumption by gender and social class across the country [4].

Smoking patterns in Nepal not only demonstrate the high smoking prevalence rates but also reveal that the individual risk behaviors may reflect the structural vulnerabilities that enhance smoking exposure and make it more difficult to quit. There are also socioeconomic factors, such as education, wealth status, and occupation, that stratify smoking patterns [5] and indicate that these are social determinants that influence smoking behavior. Lower levels of education and manual labor occupations have a higher prevalence of smoking, emphasizing that individual choices are influenced by broader social context [6].

Both globally and within Nepal, smoking has long been recognized as a national and international health problem [7]. At the global level, the World Health Organization Framework Convention on Tobacco Control (WHO FCTC) serves as the international treaty to protect future generations from the devastating effect, health‐wise, socially, environmentally, and economically, of exposure to smoking [8]. The FCTC requires its parties to implement a comprehensive and integrated approach to the prevention of smoking or addiction through various methods, such as reducing demand, controlling supply, advertising restrictions, health warnings, and providing additional avenues for preventing exposure to second‐hand smoke, among other strategic interventions. In addition to this, the MPOWER package provides further clarification of the FCTC's objective through evidence‐based interventions to effectively implement the FCTC objectives, and it has been recognized as best practice for national tobacco control bylaws [9].

In Nepal, national tobacco control policy has been developed through domestic laws aligned with these international frameworks. These laws seek to reduce consumption and control and regulate the importation, manufacture, sales, and distribution of tobacco products by creating smoke‐free environments, advertising and promoting, and placing health warnings on tobacco product packaging [10]. Although there is evidence from the policy briefs and evaluations that also shows very limited enforcement and compliance with the laws in Nepal, major challenges particularly in enforcing restrictions on sales to minors and pregnant women, regulating tobacco sales near education and health institutions, maintaining licensed tobacco sales, and ensuring smoke‐free environments with adequate cessation support [7, 10]. Enforcement and compliance of these laws have not been strong, as in Nepal's policies of smoke‐free environments and low cessation support [10]. Therefore, the overall effect of the tobacco control laws for the protection of public health is severely impaired [7].

The objective of this study is to examine the extent of gender differences in smoking patterns among adults aged 15–49 years in Nepal using the data obtained from the NDHS 2022. Specifically, this study estimates the prevalence of smoking patterns and identifies a set of geographical/residential, socioeconomic, demographic, and contextual factors associated with smoking behaviors. By identifying population groups at higher risk of smoking, this study aims to support the design and implementation for targeted policy and strategies in an equitable manner, as well as to provide evidence to support tobacco control policy development at both national and international levels.

## 2 Methods

### 2.1 Study Design and Data Sources

The study analyzed data collected from the NDHS 2022, utilizing a cross‐sectional analytical study design. The NDHS is a nationally representative household survey carried out by the Ministry of Health Population of Nepal in association with New Era and ICF as a part of the global Demographic Health Survey (DHS) program [4]. The survey used an updated version of the frame from the Nepal Population and Housing Census (NPHC) provided by the National Statistical Office [4]. The survey used a two‐stage stratified cluster sampling design. Data were collected using standardized and pre‐tested questionnaires through face‐to‐face interviews across all seven provinces, three ecological regions, and two areas of residence of Nepal.

### 2.2 Study Population and Sample Size

A survey of the DHS 2022 in Nepal, which included women and men in the age group of 15–49 years, was undertaken on the survey of both sexes. A total of 14,845 eligible women and 4913 eligible men were included in these analyses. The number of sample populations who smoke was 566 (3.8%) among women and 1,374 (28.0%) among men. The sample was limited to individuals aged 15–49 years, consistent with the NDHS 2022 standard DHS framework for adult behavioral indicators, to ensure comparability and consistency of estimates. The survey used a nationally representative sample for the country; therefore, it provides an accurate estimate of the demographics across the two areas of residence, three ecological regions and seven provinces.

### 2.3 Dependent Variable

In this study, the dependent variable is defined as women and men who consumed cigarettes as smoking, coded as a binary outcome, with 1 indicating “Yes” for every day and some days (frequency of smoking cigarettes) and 0 indicating “No” for those who do not smoke [4]. Other local forms of smoking, such as bidis, hookahs, and other tobacco products, were not included in the analysis.

### 2.4 Independent Variables

Four groups were formed on the basis of independent variables, such as geographical/residential factors (place of residence, ecological region, and province), socioeconomic and demographic factors (religion, caste/ethnic group, marital status, education level, occupation, currently working status, wealth index, age in groups, sex of the household head, and consumption of alcohol), information and awareness factors (internet use, media exposure, and health program exposure), and healthcare service and utilization factors (decision making for health care, self‐reported health status, anxiety status [GAD: generalized anxiety disorder], depression status [PHQ: Patient Health Questionnaire], and covered by health insurance). Some of the variables, such as place of residence, ecological region, province, currently working status, wealth index, sex of the household head, and covered by health insurance, were used as defined in the original DHS dataset, whereas other remaining variables were adjusted as outlined in Table 1.

**TABLE 1:** Adjusted category of independent variables.

Variable	Category	Adjusted category
Religion	Hindu	Hindu
	Buddhist	Buddhist
	Muslim, Kirat, Christian, and others	Muslim, Kirat, Christian, and others
Caste and ethnic group	Hill Brahmin and Hill Chhetri	Brahmin/Chhetri
	Hill Dalit and Tarai Dalit	Dalit
	Newar, Hill Janajati, and Tarai Janajati	Janajati
	Tarai Brahmin/Chhetri, other Tarai caste, Muslim, and other	Madhesi, Muslim, and others
Marital status	Never in union	Never married
	Married and Living with partner	Married/Living with partner
	Widowed, divorced, and no longer living together/separated	Widow/divorced/Separated
Education level	No education	No education
	Basic	Basic
	Secondary and higher	Secondary and higher
Occupation	Professional/technical/managerial, clerical, sales and service, skilled manual, and unskilled manual	Nonagriculture
	Agriculture	Agriculture
	Not working and didn't work in the last 12 months	Not working
Age in groups	15, 16, 17, 18, 19	<20
	20, 21, 22, 23, 24, 25, 26, 27, 28, 29, 30, 31, 32, 33, 34	20–34
	35, 36, 37, 38, 39, 40, 41, 42, 43, 44, 45, 46, 47, 48, 49	35–39
Consumption of any alcohol in the past month	Did not have even one drink	No
	Every day/almost every day and 1–30 times in the past month	Yes
Decision making for health care	Respondent alone	Respondent alone
	Respondent and husband/partner and respondent and other person	Respondent, husband/partner, and others
	Husband/partner alone	Husband/partner alone
	Someone else and other	Someone else and others
Self‐reported health status	Very good and good	Very good/good
	Moderate	Moderate
	Very bad and bad	Very bad/bad
Anxiety status (GAD)	Mild anxiety (GAD Score: 0–5)	No
	Moderate and severe anxiety (GAD Score: 6–21)	Yes
Depression status (PHQ)	Minimal depression (PHQ Score: 0–4)	No
	Mild, moderate, moderately severe, and severe depression symptoms (PHQ Score: 5–27)	Yes

Abbreviations: GAD, generalized anxiety disorder; PHQ, Patient Health Questionnaire.

Furthermore, women were classified as having mass media exposure if they reported reading newspaper or magazine, frequency of watching television, and frequency of listening to radio, almost every day, at least once a week, and less than once a week. Participation in any of these categories was coded as “yes,” whereas all others were coded as “no.” Additionally, women were categorized as internet users if they reported using yes, either in last 12 months and yes or before last 12 months; otherwise, they were classified as nonusers. Similarly, women were categorized as having health program exposure if they had heard or seen at least one of the eight specific health‐related programs on the radio or television; otherwise, they were classified as having no exposure.

### 2.5 Data and Statistical Analyses

Stata 13.0 was used for processing and analyzing the data [11]. Along with the “svyset” command for sampling weight, clustering, and stratification considerations were included in the complex sample analysis using Stata in order to calculate accurate estimation and significance tests. The variables that were found to be statistically significantly associated with the dependent variable by chi‐square tests were further assessed by multivariable binary logistic regression to examine their relationships. Variance inflation factor (VIF) tests were performed to check for multicollinearity prior to running a binary logistic regression, and no multicollinearity issues (all VIFs < 2.0) were found among the independent variables [12] for women and men except in marital status, which was excluded from the multivariable binary logistic regression analyses. Results from the logistic regression analyses were presented as adjusted odds ratio (AOR) with 95% CI, where *p* < 0.05 is considered statistically significant. Because of multiple covariates and outcomes were analyzed, there is a possibility of inflated Type I error due to multiple testing, which should be considered when interpreting the findings.

## 3 Results

Table 2 showed the percentage distribution of women and men who had smoked, disaggregated by various factors, such as geographical/residential factors, socioeconomic and demographic factors, information and awareness factors, and health care and service utilization factors, presented with study variables used for Nepal in DHS datasets. Prior to conducting multivariable binary logistic regression, bivariate associations between smoking patterns and explanatory variables were examined using the chi‐square test. Among women, all variables were significantly associated with smoking patterns except health program exposure, which showed no significant association among women. Among men, six variables, including place of residence, ecological region, wealth index, sex of the household head, media exposure, and decision making for health care, were not significantly associated with smoking patterns in the bivariate analysis, whereas the remaining variables showed associations.

**TABLE 2:** Percentage distribution of women and men by smoking pattern in Nepal by study variables, Demographic Health Survey (DHS) 2022.

	Smoking pattern							
	Women				Men			
Categories	Number	Percent (95% CI)	Total	*χ* ^2^ (*p* value)	Number	Percent (95% CI)	Total	*χ* ^2^ (*p* value)
**Geographical/Residential factors**								
Place of residence								
Urban	326	3.2 (2.7–3.8)	10,178	0.000	970	28.0 (25.9–30.3)	3462	0.925
Rural	241	5.2 (4.4–6.0)	4667		404	27.9 (25.4–30.5)	1451	
Ecological region								
Mountain	57	7.2 (5.1–10.1)	791	0.000	80	31.5 (27.4–36.0)	255	0.433
Hill	302	5.1 (4.4–6.0)	5872		540	27.3 (24.8–30.0)	1973	
Tarai	207	2.5 (2.0–3.2)	8182		754	28.1 (25.7–30.6)	2685	
Province								
Koshi	82	3.3 (2.5–4.3)	2493	0.000	291	33.0 (29.6–36.6)	882	0.000
Madhesh	34	1.1 (0.8–1.7)	3010		206	20.7 (17.9–23.8)	997	
Bagmati	181	5.9 (4.6–7.6)	3062		382	31.5 (27.1–36.2)	1214	
Gandaki	46	3.3 (2.4–4.5)	1401		102	26.4 (21.7–31.9)	387	
Lumbini	88	3.3 (2.3–4.6)	2691		227	28.0 (24.0–32.3)	812	
Karnali	75	8.3 (6.8–10.0)	909		63	23.8 (19.6–28.5)	266	
Sudurpaschim	60	4.7 (3.7–6.0)	1279		101	28.6 (23.8–33.9)	355	
**Socioeconomic and demographic factors**								
Religion								
Hindu	448	3.6 (3.2–4.1)	12,374	0.000	1105	27.5 (25.7–29.3)	4025	0.017
Buddhist	68	7.0 (5.1–9.5)	970		139	35.9 (29.2–43.1)	389	
Muslim, Kirat, Christian, and others	50	3.4 (2.4–4.7)	1500		129	25.9 (21.8–30.5)	499	
Caste and ethnic group								
Brahmin/Chhetri	139	3.4 (2.8–4.0)	4152	0.000	305	24.8 (21.7–28.1)	1232	0.000
Dalit	113	5.1 (3.9–6.5)	2240		224	34.0 (29.4–38.9)	658	
Janajati	287	5.3 (4.5–6.2)	5428		614	32.9 (30.4–35.5)	1869	
Madhesi, Muslim, and others	27	0.9 (0.5–1.4)	3024		231	20.0 (17.5–22.7)	1154	
Marital status								
Never married	34	1.1 (0.7–1.6)	3203	0.000	406	23.0 (20.7–25.5)	1768	0.000
Married/Living with partner	482	4.3 (3.8–4.9)	11,180		947	30.5 (28.5–32.6)	3101	
Widow/divorced/separated	51	11.0 (8.2–14.6)	462		21	46.9 (31.6–62.8)	44	
Education level								
No education	343	9.0 (8.0–10.3)	3796	0.000	123	31.3 (26.0–37.1)	393	0.000
Basic	138	3.0 (2.3–3.9)	4595		676	35.6 (33.0–38.3)	1898	
Secondary and higher	85	1.3 (1.0–1.7)	6454		575	21.9 (20.1–23.9)	2621	
Occupation								
Nonagriculture	133	3.7 (2.9–4.7)	3552	0.000	991	32.2 (30.0–34.5)	3080	0.000
Agriculture	375	5.3 (4.6–6.0)	7131		293	25.3 (22.4–28.5)	1156	
Not working	58	1.4 (1.0–1.9)	4147		89	13.3 (10.3–17.0)	672	
Currently working status								
No	125	2.1 (1.7–2.6)	6007	0.000	240	21.4 (18.5–24.7)	1119	0.000
Yes	441	5.0 (4.4–5.7)	8838		1134	29.9 (28.0–31.9)	3794	
Wealth index								
Poorest	206	7.8 (6.7–9.1)	2628	0.000	224	29.8 (26.5–33.3)	751	0.128
Poorer	104	3.6 (3.0–4.4)	2857		285	30.5 (27.2–34.1)	933	
Middle	105	3.5 (2.6–4.7)	3028		266	27.8 (24.6–31.3)	957	
Richer	95	3.0 (2.2–3.9)	3197		317	27.9 (24.6–31.5)	1135	
Richest	57	1.8 (1.3–2.5)	3135		282	24.8 (21.7–28.2)	1137	
Age in groups								
<20	19	0.7 (0.4–1.3)	2643	0.000	163	16.6 (13.7–19.9)	985	0.000
20–34	152	2.1 (1.6–2.7)	7216		727	33.2 (30.6–36.0)	2190	
35–49	395	7.9 (7.0–8.9)	4986		484	27.8 (25.4–30.3)	1739	
Sex of household head								
Male	324	3.3 (2.9–3.8)	9745	0.001	1136	28.4 (26.5–30.3)	4003	0.272
Female	243	4.8 (4.1–5.6)	5100		238	26.1 (22.6–29.9)	910	
Consumption of any alcohol in the past month								
No	354	2.7 (2.4–3.0)	13,226	0.000	468	16.5 (14.8–18.4)	2836	0.000
Yes	212	13.1 (10.9–15.7)	1619		906	43.6 (41.0–46.3)	2077	
**Information and awareness factors**								
Internet use
No	354	7.1 (6.2–8.0)	4993	0.000	335	30.9 (28.0–34.1)	1084	0.017
Yes	213	2.2 (1.8–2.6)	9852		1038	27.1 (25.3–29.0)	3829	
Media exposure								
No	149	4.7 (3.9–5.8)	3135	0.019	201	26.9 (22.1–32.2)	747	0.628
Yes	418	3.6 (3.1–4.1)	11,710		1173	28.2 (26.5–29.9)	4166	
Health program exposure								
No	438	3.9 (3.4–4.4)	11,341	0.614	1049	30.1 (28.0–32.3)	3481	0.000
Yes	128	3.7 (3.0–4.5)	3504		325	22.7 (20.5–25.1)	1432	
**Healthcare and service utilization factors**								
Decision making for health care								
Respondent alone	139	5.6 (4.6–6.8)	2487	0.000	472	31.2 (28.4–34.2)	1512	0.893
Respondent, husband/partner, and others	235	4.2 (3.6–5.0)	5531		381	30.0 (27.0–33.0)	1270	
Husband/partner alone	95	4.2 (3.4–5.1)	2297		48	28.8 (21.8–37.0)	168	
Someone else and others	12	1.4 (0.8–2.5)	865		46	30.4 (23.0–39.0)	152	
Total^a^	482	4.3 (3.8–4.9)	11,180		947	30.5 (28.5–32.6)	3101	
Self‐reported health status								
Very good/Good	132	2.7 (2.2–3.2)	4993	0.000	589	25.2 (23.0–27.5)	2336	0.002
Moderate	333	4.0 (3.4–4.5)	8423		705	30.0 (27.5–32.7)	2348	
Very bad/Bad	101	7.1 (5.7–8.7)	1429		80	34.9 (28.3–42.1)	230	
Anxiety status (GAD)								
No	190	3.3 (2.8–3.9)	5785	0.000	1161	26.6 (25.0–28.4)	4358	0.000
Yes	104	6.4 (5.1–7.9)	1626		213	38.3 (33.3–43.7)	555	
Total^b^	294	4.0 (3.4–4.6)	7410					
Depression status (PHQ)								
No	198	3.4 (2.8–4.0)	5847	0.001	1180	27.0 (25.3–28.8)	4367	0.000
Yes	96	6.1 (5.0–7.5)	1564		194	35.4 (31.0–40.1)	546	
Total^b^	294	4.0 (3.4–4.6)	7410					
Covered by health insurance								
No	528	4.0 (3.6–4.5)	13,070	0.001	1221	28.6 (26.8–30.5)	4261	0.032
Yes	39	2.2 (1.5–3.2)	1775		153	23.5 (19.6–28.0)	652	
Nepal	566	3.8 (3.4–4.3)	14,845		1374	28.0 (26.3–29.7)	4913	

Abbreviations: GAD, generalized anxiety disorder; PHQ, Patient Health Questionnaire.

^a^Different total number (for women: 11,180 and for men: 3101).

^b^Different total number (for women: 7410).

Overall, men revealed a higher prevalence of smoking patterns (28.0%, 95% CI: 26.3%–29.7%) compared to women (3.8%, 95% CI: 3.4%–4.3%) presented in Table 2. Significant provincial variation was observed for both women and men (*p* < 0.001). Among women, smoking prevalence was found highest in Karnali province (8.3%) and lowest in Madhesh province (1.1%), but among men, the highest prevalence was reported in Koshi (33.0%) and Bagmati (31.5%), whereas Madhesh showed comparatively lower prevalence (20.7%). Furthermore, smoking patterns showed significant by caste/ethnic groups (*p* < 0.001), by caste/ethnic groups Dalit and Janajati groups were observed to have higher prevalence than Brahmin/Chhetri and Madhesi, Muslim, and other groups for both sexes. Education showed a strong inverse relationship with smoking patterns; women with no education had the highest prevalence (9.0%), compared to women with secondary or higher education (1.3%). A similar gradient was also observed among men: 21.9% for men with no education and 31.3% for men with secondary or higher education. Smoking pattern was clearer among currently working women and men adults than those who were not working (women: 5.0% vs. 2.1%; men: 29.9% vs. 21.4%; *p* < 0.001). Age was also another significant factor (*p* < 0.001) associated with smoking patterns, although the relationship was not strictly across all age groups. Among women, smoking prevalence was highest (7.9%) in the 35–49 age group, whereas among men it was highest (33.2%) in the 20–34 age group. Alcohol consumption was also observed as another strong associator with smoking patterns. Women who consumed alcohol had nearly five times greater smoking prevalence (13.1%) compared to nonconsumers (2.7%) of alcohol, whereas men who consumed alcohol had a higher prevalence (43.6%) than nonconsumers (16.5%) of alcohol (*p* < 0.001).

Table 3 presents multivariable binary logistic regression results identifying factors associated with smoking patterns among adults aged 15–49 years in Nepal. After adjustment, the province showed significance in both women and men. Compared to Madhesh province, women residing in Bagmati (AOR: 2.669; 95% CI: 1.020–6.981; *p* = 0.045) and Karnali (AOR: 3.144; 95% CI: 1.360–7.268; *p* = 0.007) had significantly higher odds of smoking patterns. Among men, smoking patterns were significantly higher in Koshi (AOR: 1.881; 95% CI: 1.375–2.573; *p* = 0.000), Bagmati (AOR: 1.505; 95% CI: 1.067–2.123; *p* = 0.000), and Sudurpaschim (AOR: 1.453; 95% CI: 1.017–2.078; *p* = 0.000), underscoring strong contextual and regional reflection on smoking patterns.

**TABLE 3:** Factors associated with smoking pattern by gender in Nepal with study variables.

Categories	Smoking pattern			
	Women (AOR)	*p* value	Men (AOR)	*p* value
**Geographical/Residential factors**				
Place of residence			#	
Urban	Ref. (1)			
Rural	1.127 (0.814–1.562)	0.471		
Ecological region			#	
Mountain	Ref. (1)			
Hill	0.696 (0.432–1.120)	0.135		
Tarai	1.069 (0.584–1.957)	0.827		
Province				
Koshi	1.161 (0.498–2.707)	0.728	1.881 (1.375–2.573)^***^	0.000
Madhesh	Ref. (1)		Ref. (1)	
Bagmati	2.669 (1.020–6.981)^*^	0.045	1.505 (1.067–2.123)^*^	0.020
Gandaki	1.797 (0.723–4.462)	0.206	1.281 (0.869–1.891)	0.211
Lumbini	1.222 (0.588–2.543)	0.590	1.280 (0.915–1.789)	0.149
Karnali	3.144 (1.360–7.268)^**^	0.007	1.226 (0.841–1.788)	0.289
Sudurpaschim	1.756 (0.851–3.623)	0.127	1.453 (1.017–2.078)^*^	0.040
**Socioeconomic and demographic factors**				
Religion				
Hindu	Ref. (1)		Ref. (1)	
Buddhist	1.280 (0.724–2.264)	0.394	1.122 (0.774–1.625)	0.544
Muslim, Kirat, Christian, and others	1.114 (0.634–1.956)	0.708	1.143 (0.835–1.563)	0.403
Caste and ethnic group				
Brahmin/Chhetri	3.897 (1.452–10.455)^**^	0.007	1.458 (1.071–1.985)^*^	0.017
Dalit	3.501 (1.426–8.596)^**^	0.006	1.402 (1.034–1.900)^*^	0.030
Janajati	4.080 (1.604–10.378)^**^	0.003	1.255 (0.941–1.673)	0.122
Madhesi, Muslim, and others	Ref. (1)		Ref. (1)	
Education level				
No education	3.705 (2.091–6.565)^***^	0.000	1.704 (1.239–2.343)^**^	0.001
Basic	1.542 (0.841–2.828)	0.161	1.786 (1.503–2.122)^***^	0.000
Secondary and higher	Ref. (1)		Ref. (1)	
Occupation				
Nonagriculture	0.857 (0.403–1.823)	0.687	2.096 (1.430–3.072)^***^	0.000
Agriculture	0.782 (0.407–1.504)	0.461	1.598 (1.055–2.421)^*^	0.027
Not working	Ref. (1)		Ref. (1)	
Currently working status				
No	1.073 (0.642–1.792)	0.788	1.346 (1.034–1.752)^*^	0.027
Yes	Ref. (1)		Ref. (1)	
Wealth index				
Poorest	Ref. (1)		#	
Poorer	0.555 (0.351–0.878)^*^	0.012		
Middle	0.814 (0.503–1.319)	0.403		
Richer	0.486 (0.252–0.940)^*^	0.032		
Richest	0.312 (0.138–0.706)	0.005		
Age in groups				
<20	Ref. (1)		Ref. (1)	
20–34	7.849 (1.599–38.519)^*^	0.011	1.597 (1.189–2.146)^**^	0.002
35–49	18.258 (3.716–89.706)^***^	0.000	1.069 (0.785–1.456)	0.669
Sex of household head			***#***	
Male	0.817 (0.589–1.133)	0.224		
Female	Ref. (1)			
Consumption of any alcohol in the past month				
No	Ref. (1)		Ref. (1)	
Yes	2.808 (1.835–4.298)^***^	0.000	3.236 (2.737–3.826)^***^	0.000
**Information and awareness factors**				
Internet use	Ref. (1)		Ref. (1)	
No	1.051 (0.723–1.527)	0.795	0.978 (0.809–1.182)	0.818
Yes				
Media exposure			***#***	
No	0.963 (0.662–1.399)	0.841		
Yes	Ref. (1)			
Health program exposure	***#***			
No			1.427 (1.198–1.700)^***^	0.000
Yes			Ref. (1)	
**Healthcare and service utilization factors**				
Decision making for health care			***#***	
Respondent alone	1.532 (0.993–2.363)	0.054		
Respondent, husband/partner, and others	0.981 (0.685–1.405)	0.918		
Husband/partner alone	Ref. (1)			
Someone else and others	1.160 (0.471–2.857)	0.746		
Self‐reported health status				
Very good/Good	Ref. (1)		Ref. (1)	
Moderate	0.851 (0.609–1.188)	0.342	1.154 (0.975–1.367)	0.096
Very bad/Bad	1.165 (0.738–1.838)	0.512	1.420 (0.986–2.045)	0.060
Anxiety status (GAD)				
No	Ref. (1)		Ref. (1)	
Yes	1.268 (0.805–1.999)	0.305	1.451 (1.115–1.889)^**^	0.006
Depression status (PHQ)				
No	Ref. (1)		Ref. (1)	
Yes	1.212 (0.786–1.870)	0.384	1.069 (0.837–1.365)	0.594
Covered by health insurance				
No	1.436 (0.798–2.584)	0.227	1.289 (0.981–1.694)	0.068
Yes	Ref. (1)		Ref. (1)	

*Note:* Ref.: reference category. #: not significant in bivariate analysis (Chi‐square test).

Abbreviations: AOR, adjusted odds ratio; GAD, generalized anxiety disorder; PHQ, Patient Health Questionnaire.

* *p* < 0.050.

** *p* < 0.010.

*** *p* < 0.001.

Women from Brahmin/Chhetri (AOR: 3.897; 95% CI: 1.452–10.455; *p* = 0.007), Dalit (AOR: 3.501; 95% CI: 1.426–8.596; *p* = 0.006), and Janajati (AOR: 4.080; 95% CI: 1.604–10.378; *p* = 0.003) communities had significantly higher odds of smoking compared to the Madhesi, Muslim, and others groups. Among men, higher odds were found among Brahmin/Chhetri (AOR: 1.458; 95% CI: 1.071–1.985; *p* = 0.017) and Dalit (AOR: 1.402; 95% CI: 1.034–1.900; *p* = 0.030) communities’ groups, indicating continuous caste and ethnic group‐based discrepancies in smoking behavior.

Women with no education had nearly four times higher odds of smoking (AOR: 3.705; 95% CI: 2.091–6.565; *p* = 0.000) compared to women with secondary or higher education. Similarly, for men with no education (AOR: 1.704; 95% CI: 1.239–2.343; *p* = 0.001) and basic education (AOR: 1.786; 95% CI: 1.503–2.122; *p* = 0.000) also showed higher odds of smoking. In terms of occupation, men from nonagricultural (AOR: 2.096; 95% CI: 1.430–3.072; *p* = 0.033) and agricultural (AOR: 1.598; 95% CI: 1.055–2.421; *p* = 0.027) work were significantly observed to be more likely to smoke than those who were not working. Women aged 20–34 (AOR: 7.849; 95% CI: 1.599–38.519; *p* = 0.011) and 35–49 years (AOR: 18.258; 95% CI: 3.716–89.706; *p* = 0.000) have significantly higher odds of smoking compared with women aged <20 years, whereas the men 20–34 age group (AOR: 1.597; 95% CI: 1.189–2.148; *p* = 0.002) also showed significant and observed higher odds of smoking compared to men aged<20 years. The exceptionally high AOR (18.258) among women 35–49 years may be partly influenced by the very low smoking prevalence in the reference group of women aged below 20 years (0.7%). Furthermore, in terms of alcohol consumption, women who consumed alcohol showed nearly three times higher odds (AOR: 2.808; 95% CI: 1.835–4.298; *p* = 0.000) of smoking, whereas men who consumed alcohol illustrated more than threefold higher odds (AOR: 3.236; 95% CI: 2.727–3.826; *p* = 0.000) compared to nonconsumers of alcohol.

## 4 Discussion

The findings of this study indicate that in Nepal, there exists a complexly structured and distinctly gendered pattern in terms of patterns of smoking, reinforcing the need for separate analysis of women and men to identify the different geographical/residential, socioeconomic and demographic, and health care and service utilization factors that influence smoking behaviors among the groups. The marked gender differences observed between significant predictors indicate that the circumstances under which women and men smoke are also fundamentally distinct.

The study provides evidence that smoking among adults in Nepal is not only an outcome of individual choice but is deeply embedded within a structural and sociocultural context. The higher odds of smoking among women in Karnali and Bagmati are especially noticeable, indicating uneven geographical development and limited access to health education and awareness [13, 14]. Similarly, among men, Koshi, Bagmati, and Sudurpaschim provinces were observed to have higher odds, aligning with studies highlighting provincial differences in substance use due to migration, trade routes, and localized social practices [15, 16]. Structural interventions should focus on high‐risk regions, particularly Karnali province for women and Koshi province for men, as these regions exhibited the highest regional disparities in smoking prevalence. These provincial patterns show the role of regional context and uneven sociocultural disparities in shaping smoking behaviors, calling for a context‐sensitive and decentralized smoking control program.

Caste and ethnic groups have emerged as significant predictors among women. The Brahmin/Chhetri, Dalit, and Janajati women have markedly higher odds of smoking compared to Madhesi, Muslim, and other caste and ethnic groups, indicating that smoking is embedded in ethnic and social identity practices, ritual uses or is a way to cope with structural marginalization [17]. For men, there is also an association, and it follows a similar gradient, indicating casted‐based social behaviors influence smoking behaviors among men [18, 19]. In some Nepalese communities, particularly among certain Janajati ethnic and caste groups who have a higher AOR, smoking is traditionally associated with cultural rituals, social gatherings, and customary practices [20], which may contribute to the higher prevalence of smoking among these populations. The educational background of the respondents showed a beneficial influence on health outcomes and was consistent with the global finding that increased levels of education increase health literacy and the likelihood of lower levels of hazardous behaviors like smoking [21]. Additionally, there is a significant association between no education and the smoking behavior of women and men, demonstrating the dual vulnerability that adults face in both gender and education, see also [22]. Given that those who lack access to education and awareness, any efforts to control smoking use patterns will need to ensure that they also invest in awareness through education. These patterns show that education and caste and ethnic group are key structural determinants beyond individual choice of smoking patterns.

Age, especially the significantly higher likelihood of smoking in women aged between 35 and 49 years, indicates cumulative exposure to structural disadvantage across the lifespan [23, 24]. Furthermore, the higher smoking prevalence among men aged 20–34 years may be associated with greater peer influence [25], social acceptance of smoking, occupational stress [26, 27], and increased exposure to smoking environments [28] during early adulthood among men, as reported in previous studies.

Occupation status emerged as a significant predictor among men; individual occupations in nonagriculture and agriculture were more likely to smoke compared to men who were not working at the time of the survey. Working status may increase social exposure, contacts, economic financial capability, and independence, which may lead to men's access to and smoking behavior [28]. However, this relationship may also reflect coping with work‐related stress and changing gender norms as men enter the workforce [26]. This smoking pattern may reflect both cumulative exposure to sociocultural smoking practices and increased social autonomy in working occupations [26]. These findings highlight the need for implementing workplace‐focused smoking cessation and stress management programs, especially in urban industrial settings, to address higher smoking prevalence among men engaged in nonagricultural occupations. The strong association between alcohol and smoking use is consistent with the well‐established syndemic for substance use, and the combination of both increases the risk of adverse health effects [29, 30]. Additionally, this synergistic relationship indicates that integrated approaches to multiple substance use issues may provide an improved outcome compared to treating each substance separately. All these behaviors regarding smoking patterns reflect the broader sociocultural norms of context rather than isolated individual decisions for smoking.

## 5 Conclusions

Smoking among adults in Nepal is shaped by sociocultural and structural factors, including province, caste/ethnic group, education, occupation, age, and alcohol consumption pattern, rather than an individual's own behavior and choice. These findings indicate smoking patterns in Nepal, embedded within structural inequalities and sociocultural hierarchies. Smoking patterns reflect broader processes of social exclusion, regional marginalization, and educational disadvantage, with alcohol consumption patterns reinforcing behavioral grouping. Therefore, addressing smoking behavior in Nepal requires structural interventions that should prioritize high‐risk regions, particularly Karnali province for women and Koshi province for men, where the highest regional disparities in smoking prevalence were observed. The Ministry of Health should implement coordinated policies and programs that jointly address smoking control and reduction of alcohol consumption rather than treating them as separate issues. All it addresses are these underlying factors, rather than relying solely on individual behavior change, to achieve equitable and sustainable public health benefits.

## Author Contributions

**Om Chandra Thasineku**: conceptualization, methodology, data curation, formal analysis, project administration, writing – original draft, writing – review and editing, software, investigation. **Yogendra Bahadur Gurung**: conceptualization, methodology, validation, supervision, formal analysis, writing – review and editing. **Sudesh Pandit**: conceptualization, methodology, validation, investigation, supervision, writing – review and editing.

## Funding

The authors have nothing to report.

## Ethics Statement

To conduct this study, the data were provided upon request, which is publicly available, with completely anonymous responses that do not identify any individual. However, before allowing access to the datasets, we formally registered and submitted a formal request to the DHS program and the final authorization to access, download, and use the datasets. Respondents were previously anonymized during data collection to ensure confidentiality and privacy.

## Consent

As this study was based on analysis of secondary data obtained from DHS of Nepal, no additional consent was required from the participants.

## Conflicts of Interest

The authors declare no conflicts of interest.

## Data Availability

The data that support the findings of this study are openly available in Demographic and Health Survey at https://dhsprogram.com/data/dataset/Nepal_Standard‐DHS_2022.cfm?flag=1.
